# Liposarcoma of the Spermatic Cord: A Rare Case Following a Decade of Radiotherapy for Prostate Cancer

**DOI:** 10.7759/cureus.85221

**Published:** 2025-06-02

**Authors:** Malik Samardali, Jehad Samardaly, Ibrahim Shanti

**Affiliations:** 1 Internal Medicine, Marshall University Joan C. Edwards School of Medicine, Huntington, USA; 2 Internal Medicine, Jordan University of Science and Technology, Irbid, JOR

**Keywords:** liposarcoma, prostate cancer, radiotherapy, secondary malignancy, spermatic cord

## Abstract

Liposarcoma of the spermatic cord is an uncommon malignant tumor, and even rarer in the setting of prior radiotherapy for prostate cancer. It often presents as a painless inguinal or scrotal mass and is frequently mistaken for more common conditions like inguinal hernia or hydrocele. We report a case of a 75-year-old man with a history of prostate cancer treated with radiotherapy a decade ago, who presented with a painless swelling in his left groin. Initial imaging suggested an inguinal hernia, but histopathological examination revealed a poorly differentiated, high-grade liposarcoma of the spermatic cord with lymphovascular invasion and periosteal involvement. Treatment included radical orchiectomy and broad tumor excision, followed by radiation therapy. Despite this, the patient experienced a recurrence, necessitating further interventions, including coil embolization and systemic therapy. This case highlights the potential association between radiotherapy for prostate cancer and the subsequent development of secondary malignancies such as liposarcoma of the spermatic cord.

## Introduction

Liposarcoma of the spermatic cord is a very rare malignancy, with fewer than 200 cases documented in the literature [1]. It often presents as a painless, firm, nodular mass in the inguinal or scrotal region and is frequently mistaken for more common conditions such as inguinal hernia, hydrocele, or lipoma. The tumor predominantly occurs in adults, with the average age at diagnosis being 61 years [2-4]. Histopathological examination is crucial for accurate diagnosis, typically revealing poorly differentiated, dedifferentiated, or sclerosing subtypes of liposarcoma [5,6]. It has been reported that preoperative diagnosis can be aided by imaging modalities such as gray-scale sonography [7]. The etiology of liposarcoma in the spermatic cord remains unclear, but there is growing interest in potential links to prior surgical interventions and radiation therapy. Prostate cancer is a prevalent malignancy among older men, and radiotherapy is a common treatment modality [8]. However, radiotherapy is associated with an increased risk of secondary malignancies, including sarcomas [9]. The development of liposarcoma following radiotherapy for prostate cancer, although rare, raises important questions about the long-term risks of radiation therapy. We report a case of a 75-year-old man who developed a poorly differentiated, high-grade liposarcoma of the spermatic cord 10 years after receiving radiotherapy for prostate cancer. This case underscores the importance of considering secondary malignancies in patients with a history of radiotherapy and highlights the need for long-term follow-up and vigilance in monitoring for potential complications.

## Case presentation

A 75-year-old male presented with a complaint of a small, painless swelling in his left groin that had been increasing in size. His medical history was significant for coronary artery disease, treated with coronary artery bypass graft surgery, and a history of hypertension, hyperlipidemia, and type 2 diabetes mellitus. Notably, he had a history of prostate cancer, for which he underwent radiotherapy six years prior. He also had a 45-year history of smoking one pack per day.

**Figure 1:**
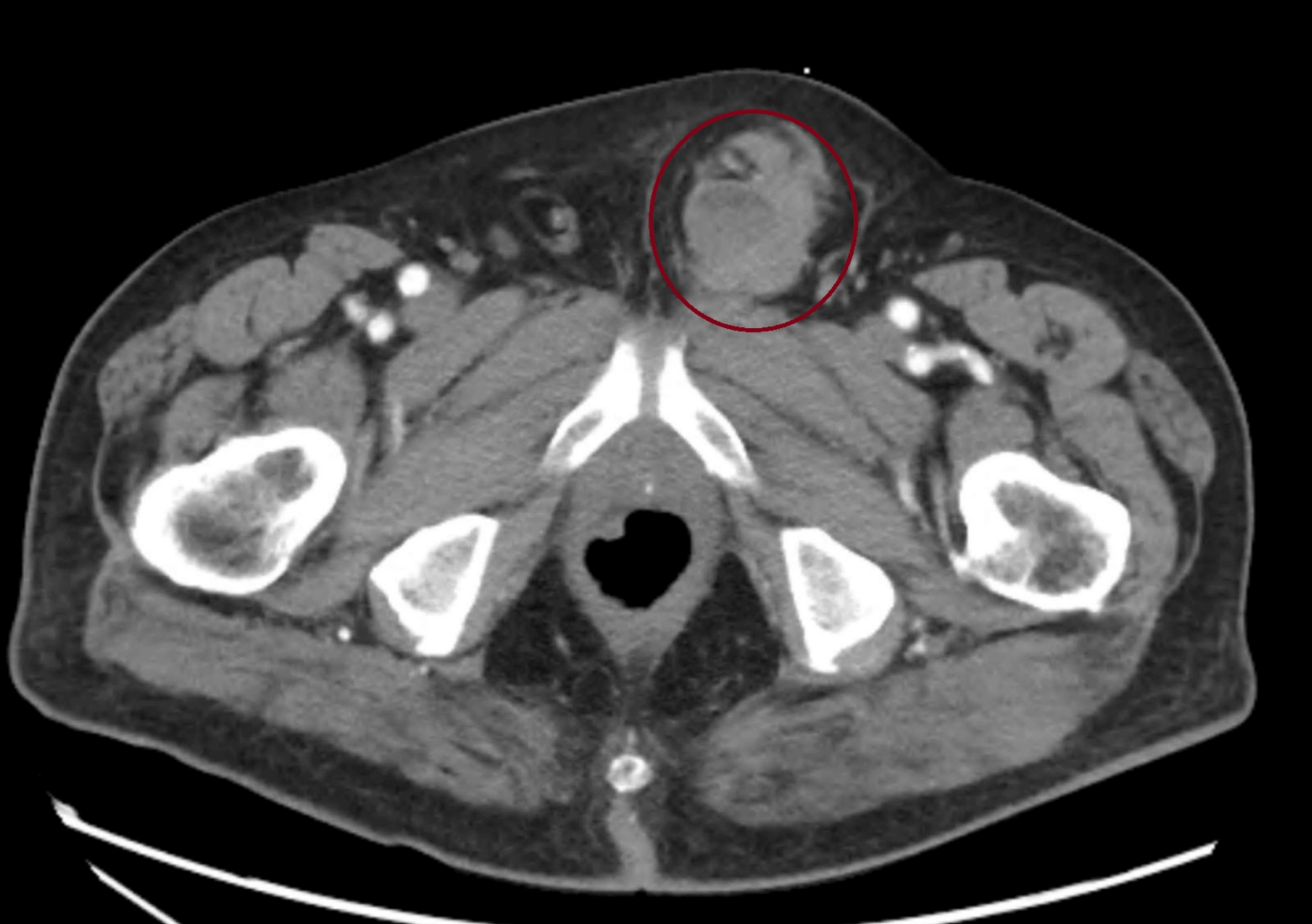
CT scan of the pelvis revealed a soft tissue mass over the left pubic tubercle, suggestive of a left inguinal hernia.

On physical examination, the patient was found to have a left inguinal lump that resembled an inguinal hernia. Abdominal and chest examinations were unremarkable. A CT scan of the pelvis revealed a soft tissue mass over the left pubic tubercle, suggestive of a left inguinal hernia (Figure 1). Scrotal ultrasound confirmed the presence of a reducible left inguinal hernia with intermittent retraction of the testis and urine passage from the bladder into the hernia.

**Figure 2:**
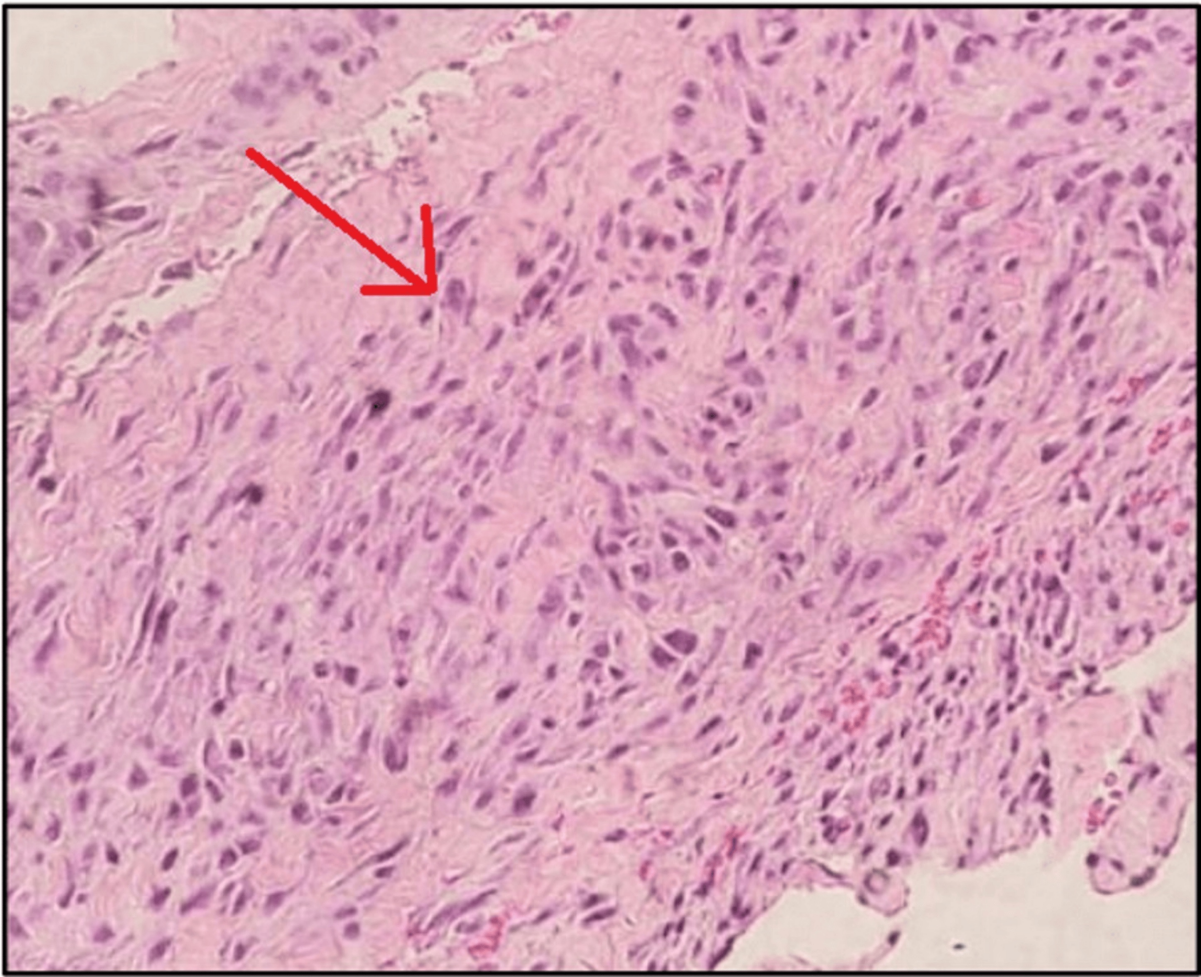
Hematoxylin and eosin (H&E) image of the biopsy of the left pubic bone showing atypical spindle cell proliferation.

Histopathological examination of the epididymis and inguinal mass revealed a poorly differentiated, high-grade liposarcoma of the spermatic cord, characterized by lymphovascular invasion and extension into surrounding veins. A biopsy of the left pubic bone showed atypical spindle cell proliferation consistent with poorly differentiated liposarcoma involving periosteal tissue (Figure 2).

The patient underwent a left orchiectomy and reconstruction of the left abdominal wall with mesh implantation. The mass attached to the left spermatic cord and left abdominal wall was excised in a wide area. Following surgery, the patient received radiation therapy for differentiated liposarcoma.

**Figure 3:**
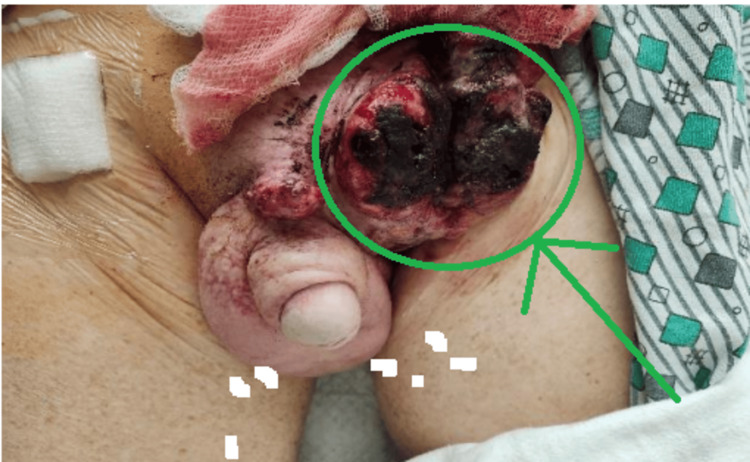
Lesion site after embolization of a branch of the left profunda femoral artery.

Twelve months later, during a follow-up visit, the patient complained of general fatigue and severe weakness. A PET scan revealed suspected metastatic disease with multiple new nodules at the lung bases. A biopsy of the lung nodules confirmed the presence of highly atypical spindle cell lesions consistent with dedifferentiated liposarcoma. The patient was started on palbociclib 125 mg, administered three weeks on and one week off, and closely monitored for his response. Despite subsequent trials of abemaciclib and pembrolizumab, the patient experienced a recurrence of the left inguinal mass, necessitating coil embolization of a branch of the left profunda femoral artery (Figure 3).

## Discussion

The case of our 75-year-old patient, who developed a liposarcoma of the spermatic cord 10 years after receiving radiotherapy for prostate cancer, underscores the potential association between radiotherapy and the subsequent development of secondary malignancies. Liposarcoma of the spermatic cord is an exceptionally rare condition, with few cases documented in the literature, making this case particularly notable [1]. Liposarcoma of the spermatic cord often presents as a painless inguinal or scrotal mass, which can be easily mistaken for more common conditions like inguinal hernia, hydrocele, or lipoma [2,3]. In our case, the patient's initial presentation was a small, painless swelling in the left groin, eventually diagnosed through imaging and histopathological examination as a poorly differentiated, high-grade liposarcoma with lymphovascular invasion and extension into surrounding veins.

The link between radiotherapy and the development of secondary malignancies, including sarcomas, has been well-documented. Studies have highlighted the increased risk of secondary cancers such as myelodysplastic syndromes, acute myeloid leukemia, and various solid tumors, including rectal, colon, and bladder cancers, following radiotherapy for prostate cancer [9-13]. The high doses of radiation required for treating prostate cancer can lead to radiation-induced complications, which potentially contribute to the development of secondary tumors [14].

The development of liposarcoma in this patient may be attributed to the high doses of radiotherapy he received as part of his prostate cancer treatment. This case aligns with previous reports suggesting that surgical trauma and radiotherapy may play roles in tumorigenesis. A similar case was reported where a poorly differentiated liposarcoma of the spermatic cord developed four years post radical retropubic prostatectomy for prostate cancer, suggesting a potential association between surgical or radiotherapy-induced trauma and liposarcoma development [15].

In managing liposarcoma of the spermatic cord, radical orchiectomy and extensive local resection remain the primary treatment modalities [16]. Despite aggressive surgical intervention, our patient experienced a recurrence, necessitating additional treatments, including coil embolization and systemic therapy with agents like palbociclib and pembrolizumab. This highlights the aggressive nature of dedifferentiated liposarcomas and the challenges in managing recurrent disease. Given the increased risk of secondary malignancies post radiotherapy, long-term monitoring is crucial for early detection and management of such complications. Our case emphasizes the need for vigilance in patients with a history of radiotherapy for prostate cancer, as secondary malignancies like liposarcoma, though rare, can significantly impact patient outcomes.

## Conclusions

This case contributes to the limited literature on secondary malignancies post radiotherapy for prostate cancer and highlights the importance of considering liposarcoma of the spermatic cord as a differential diagnosis in patients presenting with inguinal or scrotal masses, especially those with a history of radiotherapy. Long-term follow-up and comprehensive management strategies are essential for optimizing patient care and outcomes.
